# Oncogenic c-terminal cyclin D1 (*CCND1*) mutations are enriched in endometrioid endometrial adenocarcinomas

**DOI:** 10.1371/journal.pone.0199688

**Published:** 2018-07-03

**Authors:** Jia Xu, Douglas I. Lin

**Affiliations:** Beth Israel Deaconess Medical Center, Department of Pathology, Boston, MA, United States of America; University of South Alabama Mitchell Cancer Institute, UNITED STATES

## Abstract

Cyclin D1 (*CCND1*) is a core cell cycle regulator and is frequently overexpressed in human cancers, often via amplification, translocation or post-transcription regulation. Accumulating evidence suggests that mutations of the *CCND1* gene that result in nuclear retention and constitutive activation of CDK4/6 kinases are oncogenic drivers in cancer. However, the spectrum of *CCND1* mutations across human cancers has not been systematically investigated. Here, we retrospectively mined whole-exome sequencing data from 124 published studies representing up to 29,432 cases from diverse cancer types and sites of origin, including carcinoma, melanoma, sarcoma and lymphoma/leukemia, via online tools to determine the frequency and spectrum of *CCND1* mutations in human cancers and their associated clinico-pathological characteristics. Overall, in contrast to gene amplification, which occurred at a frequency of 4.8% (1,419 of 28,769 cases), *CCND1* mutations were of very low frequency (0.5%, 151 of 29,432 cases) across all cancers, but were predominantly enriched in uterine endometrioid-type adenocarcinoma (6.5%, 30 of 458 cases) in both primary tumors and in advanced, metastatic endometrial cancer samples. *CCND1* mutations in endometrial endometrioid adenocarcinoma occurred most commonly in the c-terminus of cyclin D1, as putative driver mutations, in a region thought to result in oncogenic activation of cyclin D1 via inhibition of Thr-286 phosphorylation and nuclear export, thereby resulting in nuclear retention and protein overexpression. Our findings implicate oncogenic c-terminal mutations of *CCND1* in the pathogenesis of a subset of human cancers and provide a key resource to guide future preclinical and clinical investigations.

## Introduction

Cyclin D1, encoded by the *CCND1* gene, is a core cell cycle regulator that promotes cellular proliferation and is frequently overexpressed in human cancers. Alterations of cyclin D1 resulting in nuclear protein expression is thought to promote tumorigenesis in various types of cancers[1][2], often as a result of *CCND1* gene amplification, such as in breast, lung, bladder carcinomas [3][4], or as a result of *CCND1* translocation in mantle cell lymphoma [5]. Accumulating evidence also suggests that mutations of the *CCND1* gene that result in nuclear retention and constitutive activation of CDK4/6 kinases are oncogenic drivers in cancer [6][7][8][9]. Oncogenic mutations of *CCND1* have been previously reported in endometrial and esophageal carcinomas [10][11][12], however, the spectrum of *CCND1* mutations across human cancers has not been systematically investigated.

The c-terminus of cyclin D1 contains a nuclear export signal and a phospho-degron, which is a region involved in cyclin D1 phosphorylation, nuclear export, ubiquitination and protein degradation. During the S phase of the cell cycle, phosphorylation of cyclin D1 at Thr-286 by GSK3β triggers cyclin D1 nuclear export and ubitiquin-mediated proteolysis via the 26S proteasome in the cytoplasm [13][14][15]. For this reason, mutations in the c-terminus of cyclin D1 may result in oncogenic activation of cyclin D1 via inhibition of Thr-286 phosphorylation, nuclear export and protein degradation, thereby promoting cyclin D1 nuclear accumulation [6]. Nuclear retention of cyclin D1 constitutively activates CDK4/6 kinases through the cell cycle and thereby promotes cellular proliferation and tumorigenesis [12].

Specific *CCND1* c-terminal mutations involving Thr286 and Pro287 have been previously reported in endometrial cancer, such as T286I, P287T, P287S, which resulted in nuclear accumulation of cyclin D1, gain-of-function and cellular transformation [10][11]. Endometrial carcinoma is the most frequently diagnosed type of gynecological malignancy, with ~40,000 new cases and 7,500 mortalities reported in the United States in 2008 [16]. Currently, there are no FDA-approved or NCCN-compendium listed treatments specifically for patients with *CCND1*-mutant uterine endometrioid carcinoma. However, there are clinical trials evaluating palbociclib, a CDK4/6 specific inhibitor (codenamed PD-0332991, trade name Ibrance) [2][17], including a phase 2 trial in patients with metastatic endometrial cancer (ClinicalTrials.gov Identifier: NCT02730429), underscoring the importance of identifying mechanisms of CDK4 activation in endometrial cancer.

Recently, The Cancer Genome Atlas (TCGA) project and other whole exome studies, such as MSK-IMPACT trial, have molecularly characterized many different types of tumors, including endometrial adenocarcinomas [18][19]. The TCGA Data Portal and other publicly available online data mining programs offer a host of tools that can be used to interrogate specific genetic events relative to survival and clinico-pathological characteristics, as well as to generate hypotheses for future testing on a variety of tumor types [20][21].

The goal of this study was to mine whole-exome sequencing data from published studies representing a large number of cases and a plethora of cancer diagnoses to determine the frequency and spectrum of activating *CCND1* mutations across human cancers. Here we demonstrate that activating, putative driver *CCND1* mutations were of low frequency across all cancer types, but were predominantly enriched in uterine endometrioid-type adenocarcinoma.

## Methods

### Study type and design

With approval of a BIDMC/DFCI Institutional Review Board, we performed an observational retrospective secondary data analysis of clinically-annotated multi-platform cancer–omics datasets from the Cancer Genome Atlas Project (TCGA) and other previously published cancer genomics datasets. A total of 124 published studies with sequencing data (S1 Table), representing 29,432 cases from diverse cancer types and sites of origin, such as carcinoma, melanoma, mesothelioma, germ cell tumor, sarcoma and lymphoma/leukemia, were interrogated for *CCND1* mutations. We exploited public available online tools and resources in order to data mine the cancer datasets with respect to *CCND1* alterations, to interrogate different clinic-pathological characteristics relative to genomic alterations of *CCND1*, and to confirm the effect of specific *CCND1* mutations on its biological and oncogenic function.

### Cancer cohorts, gene amplification and gene mutation

Clinical and molecular data from up to 124 previously published cancer genomics studies (S1 Table) was analyzed with respect to *CCND1* amplification and mutations via the cBio Cancer Genomics Portal (http://cbioportal.org; Memorial Sloan Kettering Cancer Center, New York, NY) [20][21]. The cohorts were queried for *CCND1* gene amplification or mutations in the cbio portal by using the advanced onco query language (*CCND1*: AMP or *CCND1*: MUT) [20][21]. In this manuscript, copy number gain was defined as gain of one gene copy number, while amplification was defined as gain of 2 or more gene copy numbers. Online analysis was performed between January 1^st^, 2015 and May 7th, 2018.

*CCND1* mutations specifically in primary endometrial carcinoma were interrogated via the cbio portal and via the advanced onco query language specifically in the endometrial carcinoma TCGA study with sequencing data (n = 248) [18]. In a similar manner, *CCND1* mutations in advanced, metastatic endometrial carcinoma samples were interrogated specifically in the MSK IMPACT clinical cohort of advanced/metastatic endometrial carcinoma cases (n = 210) via the cBio portal [19].

In the primary endometrial carcinoma TGCA cohort, the following molecular and histological subsets were also analyzed via the cBio portal and by using the advanced onco query language: 1) complete endometrioid-type tumors (n = 232), 2) copy number high tumors (n = 60), 3) copy number low tumors (n = 90), 4) MSI (hypermutated) tumors (n = 65), 5) POLE (ultramutated) tumors (n = 17), and 6) tumors with specific serous-type histology (n = 53).

### Review of pathological data

Pathology reports from the endometrial carcinoma TCGA corhort were downloaded via the cBio portal. Quality control hematoxylin and eosin (H&E) images from frozen and permanent sections from TCGA cohort were re-analyzed by a board-certified gynecologic pathologist (D.L.) to confirm histological subtype via the TCGA Biosig website hosted at Lawrence Berkeley National (http://tcga.lbl.gov:8080/biosig/tcgadownload.do), and via the Cancer Digital Slide Archive (http://cancer.digitalslidearchive.net; Emory University, Atlanta, GA). Tumor stage and overall FIGO grade were also independently re-evaluated whenever possible; however, they were deferred to the available pathology reports since only limited virtual images from each case were publicly available for review. For the MSK IMPACT cohort of advanced, metastatic endometrial cancer, histological subtype was deferred to the reported subtype since H&E images were not publicly available for re-review.

### Analysis of *CCND1* mutations

Somatic *CCND1* mutations derived from exome studies, including from TCGA and MSK IMPACT endometrial carcinoma cohorts, were retrieved via the cBio portal (http://www.cbioportal.org/public-portal/). The effect of specific somatic mutations on *CCND1* function was determined by cross-referencing them to previously published reports, wherein the effect of specific *CCND1* mutations on Cyclin D1 activation or gain-of-function was demonstrated either *in vitro* or *in vivo* [6][12][11]. In addition, effects of *CCND1* mutations were independently queried in the online Precision Oncology Knowledge Base tool (http://www.OncoKB.org), which is a publicly available, evidence-based web resource tool that annotates and curates the biologic and oncogenic effects, as well as the predictive significance of somatic molecular alterations present in patient tumors with the goals of facilitating the interpretation of genomic alterations and supporting optimal treatment decisions [22].

## Results

### Frequency of *CCND1* mutations across cancer

**Fig 1:**
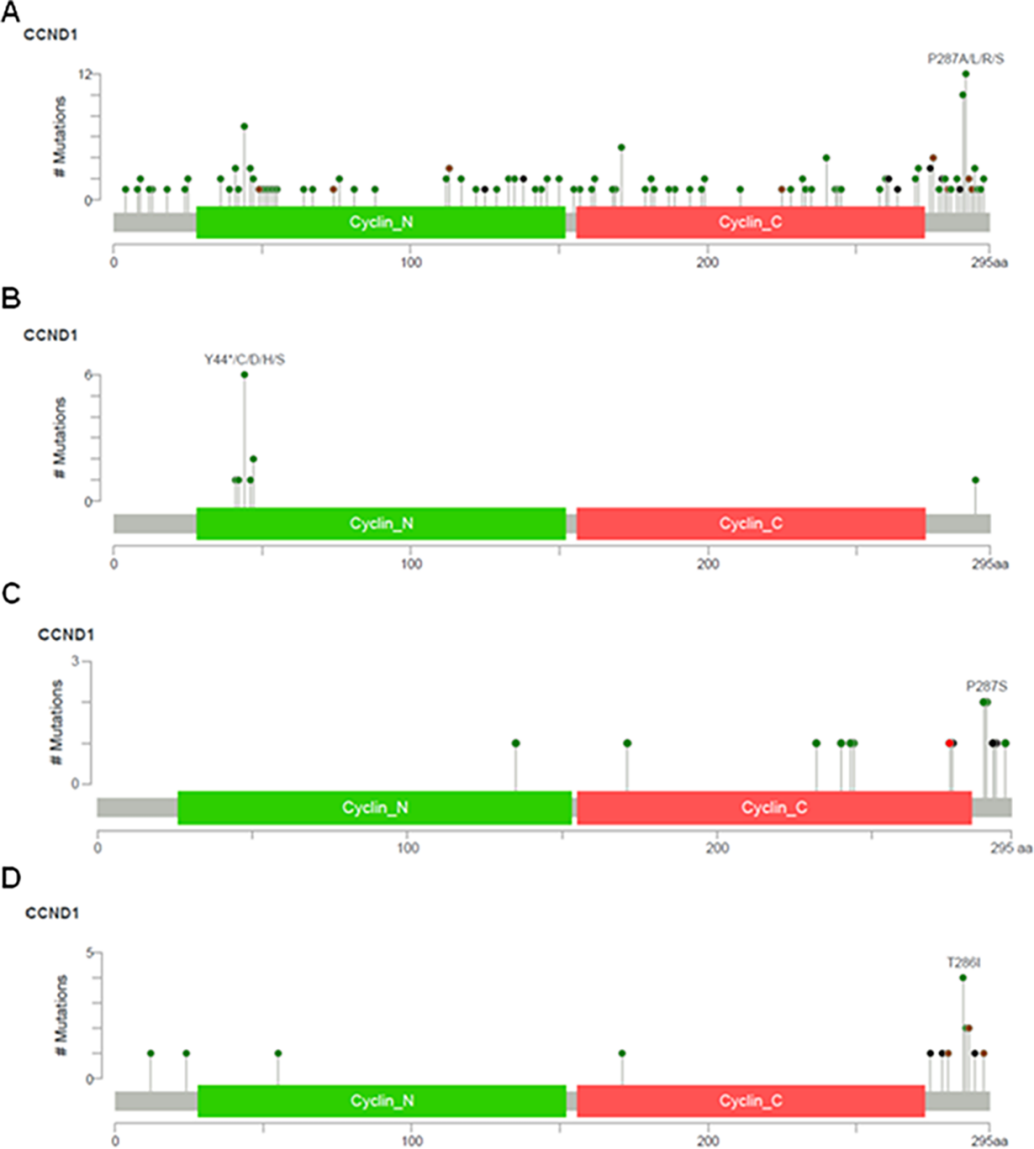
Diagrams of *CCND1* mutations. Across all human cancers analyzed (A), or specifically in the mantle cell lymphoma cohort (B), in the primary endometrial endometrioid adenocarcinoma TCGA cohort (C), and in the advanced or metastatic endometrial carcinoma MSK-IMPACT cohort (D).

To assess frequency and spectrum of *CCND1* mutations across cancer, molecular data from 124 studies (S1 Table), representing 29,432 cases/patients from diverse cancer types and sites of origin, were mined via the cBio Cancer Genomics Portal (http://cbioportal.org) [20][21]. Somatic mutation of the *CCND1* gene was seen in 0.5% (151 of 29,432) of cases spanning all regions of the cyclin D1 protein (Table 1 and Fig 1A). By combining the molecular data of studies from the same cancer subtype and site of origin, the most frequently *CCND1*-mutated cancer subtypes were mantle cell lymphoma (34.5%, 10 of 29 cases), endometrial adenocacinoma (6.5%, 30 of 458 cases), multiple-myeloma (3.3%, 7 of 205 cases), colorectal carcinoma (1.1%, 23 of 2067 cases), melanoma (1.1%, 8 of 708 cases), skin non-melanoma cancer (1.1%, 2 of 177 cases) and uterine sarcoma (1.1%, 1 of 93 cases).

**Table 1:** Exome studies containing *CCND1* mutations with respective cancer type and *CCND1* mutation frequency.

Study	Cancer type (Source, citation)	*CCND1* mut	Sample size	% mutated
1	Mantle Cell Lymphoma (IDIBIPS 2013)	10	29	34.5%
2	Melanoma (Broad/Dana Farber, Nature 2012)	2	25	8.0%
3	Endometrial Carcinoma (TCGA, Nature 2013)	15	248	6.1%
4	Glioma (MSK, Neuro Oncol 2017)	1	22	4.5%
5	Colorectal carcinoma (Genentech, Nature 2012)	3	72	4.2%
6	Skin sq. cell carcinoma (DFCI, Clin Cancer Res 2015)	1	29	3.4%
7	Multiple Myeloma (Broad, Cancer Cell 2014)	7	205	3.3%
8	Stomach Adenocarcinoma (Tokyo, Nat Genet 2014)	1	30	3.3%
9	NCI-60 Cell Lines (NCI, Cancer Res. 2012)	2	60	3.3%
10	Skin melanoma UCLA (Cell 2016)	1	38	2.6%
11	Colorectal Adenocarcinoma (DFCI 2016)	15	619	2.4%
12	Rhabdomyosarcoma (NIH, Cancer Discov 2014)	1	43	2.3%
13	Skin Cutaneous Melanoma (Broad, Cell 2012)	2	121	1.7%
14	Low-Grade Gliomas (UCSF, Science 2014)	1	61	1.6%
15	Bladder Urothelial Carcinoma (TCGA, Nature 2014)	2	130	1.5%
16	Lung Squamous Cell Carcinoma (TCGA, Nature 2012)	2	178	1.1%
17	Lung Adenocarcinoma (TCGA, Nature 2014)	2	230	0.9%
18	Breast cancer xenog (British Columbia, Nature 2014)	1	116	0.9%
19	NSCLC (TCGA 2016)	9	1144	0.8%
20	Stomach/Esophageal (TCGA)	4	518	0.8%
21	Cancer Cell Line Encyclop (Novartis/Broad, Nat 2012)	7	1020	0.7%
22	Ampulary carcinoma (BMC, 2016)	1	160	0.6%
23	Lung Adenocarcinoma (Broad, Cell 2012)	1	183	0.5%
24	MSK-IMPACT various tumor types (Nat Med 2017)	57	10945	0.5%
25	LGG-GBM (TCGA 2016)	3	812	0.4%
26	H&N Squamous Cell Carcinoma (TCGA, Nature 2015)	1	279	0.4%
27	Stomach Adenocarcinoma (TCGA, Nature 2014)	1	290	0.3%

The most commonly mutated amino acid of cyclin D1 was Proline 287, followed by Threonine 286, both of which are located in the c-terminus of cyclin D1 (Fig 1A). The third most common mutated amino acid was Tyrosine 44, which is located in the n-terminus of cyclin D1 (Fig 1A). The location of *CCND1* mutations appeared to be tissue specific. In mantle cell lymphoma and multiple myeloma, the majority of *CCND*1 mutations occurred within the amino terminal domain of cyclin D1 (S2 Table and Fig 1B), while in endometrial adenocarcinomas, and less frequently in colorectal carcinoma and melanoma, *CCND1* mutations centered on the carboxy terminal domain (Fig 1C and 1D and S1 Fig). These findings support the notion that endometrial adenocarcinomas are enriched for a unique spectrum of somatic *CCND1* alterations, in which a significant number of *CCND1* mutations occur in the c-terminal domain of cyclin D1 compared to other cancer subtypes.

### Frequency of *CCND1* amplification in cancer

Somatic *CCND1* mutations occurred less frequently than *CCND1* gene amplification. Overall, in contrast to somatic mutations, *CCND1* gene amplification occurred at a frequency of 4.8% (1,419 of 28,769 cases/patients) across all cancers and studies with gene amplification data (Table 2). By combining the molecular data of studies from the same cancer subtype and site of origin, the most frequently *CCND1*-amplified cancer subtypes were head and neck squamous cell carcinoma (27.6%, 77 of 279 cases), breast cancer (16.7%, 735 of 4406 cases), bladder cancer (6.9%, 65 of 938 cases), esophageal-gastric cancer (4.8%, 72 of 1494 cases), non-small cell lung cancer (4.6%, 168 of 3648 cases), melanoma (3.3%, 23 of 708 cases), ovarian cancer (2.5%, 14 of 551 cases), appendiceal cancer (2.5%, 2 of 79 cases), hepatobiliary cancer (2.4%, 24 of 997 cases), prostate cancer (2.4%, 54 of 2262 cases) and endometrial cancer (2.3%, 11 of 480 cases).

**Table 2:** Exome studies containing *CCND1* amplification with respective cancer type and *CCND1* amplification frequency.

Study	Cancer type (Source, citation)	*CCND1* AMP	Sample size	% amplified
1	H&N Squamous Cell Carcinoma (TCGA, Nat 2015)	77	279	27.6%
2	Meduloblastoma (Sickkids, Nature 2016)	52	213	24.4%
3	Neuroendo Prostate Cancer (Tr/Corn/Broad 2016)	23	107	21.5%
4	Breast Invasive Carcinoma (METABRIC, Nat 2012)	344	2051	16.8%
5	Breast Invasive Carcinoma (TCGA, Cell 2015)	79	505	15.4%
6	Bladder Cancer (MSKCC, J Clin Oncol 2013)	14	97	14.4%
7	NCI-60 Cell Lines (NCI, Cancer Res. 2012)	8	60	13.3%
8	Lung Squamous Cell Carcinoma (TCGA, Nat 2012)	22	178	12.4%
9	Stomach/Esophageal (TCGA)	32	265	12.1%
10	Bladder Urothelial Carcinoma (TCGA, Nat 2014)	15	128	11.7%
11	Cancer Cell Line Encyclop (Nov/Broad, Nat 2012)	112	995	11.3%
12	Prostate Adenocarcinoma (Fred Hutchinson CRC)	14	136	10.3%
13	Pancreatic Cancer (UTSW, Nat Commun 2015)	10	109	9.2%
14	Bladder Cancer (MSKCC, Eur Urol 2014)	9	109	8.3%
15	Non-small cell lung cancer (TCGA, 2016)	89	1144	7.8%
16	Metastatic prostate carcinoma (Michig, Nat 2012)	4	61	6.6%
17	Stomach Adenocarcinoma (TCGA, Nature 2014)	18	287	6.3%
18	Liver HCC (AMC, Hepatology 2014)	12	231	5.2%
19	Breast cancer xenog (British Columbia, Nat 2014)	6	116	5.2%
20	Metastatic Prostate, SU2C (Cell 2015)	7	150	4.7%
21	Lung Adenocarcinoma (TCGA, Nature 2014)	10	230	4.3%
22	MSK-IMPACT various tumor types (Nat Med 2017)	472	10945	4.3%
23	Ovarian serous carcinoma (TCGA, Nature 2011)	19	489	3.9%
24	Sarcoma (MSKCC/Broad, Nat Genet 2010)	7	207	3.4%
25	Endometrial Carcinoma (TCGA, Nature 2013)	9	363	2.5%
26	Prostate Adenocarcinoma (TCGA, Cell 2015)	7	333	2.1%
27	Adenoid Cystic Carcinoma (MSKCC, Nat Gen 2013)	1	60	1.7%
28	Lung Adenocarcinoma (Broad, Cell 2012)	3	182	1.6%
29	Glioblastoma (TCGA, Cell 2013)	3	563	0.5%
30	Acute Myeloid Leukemia (TCGA, NEJM 2013)	1	191	0.5%
31	LGG and GBM (TCGA, 2016)	2	794	0.3%

### *CCND1* mutations in primary endometrial adenocarcinomas

**Fig 2:**
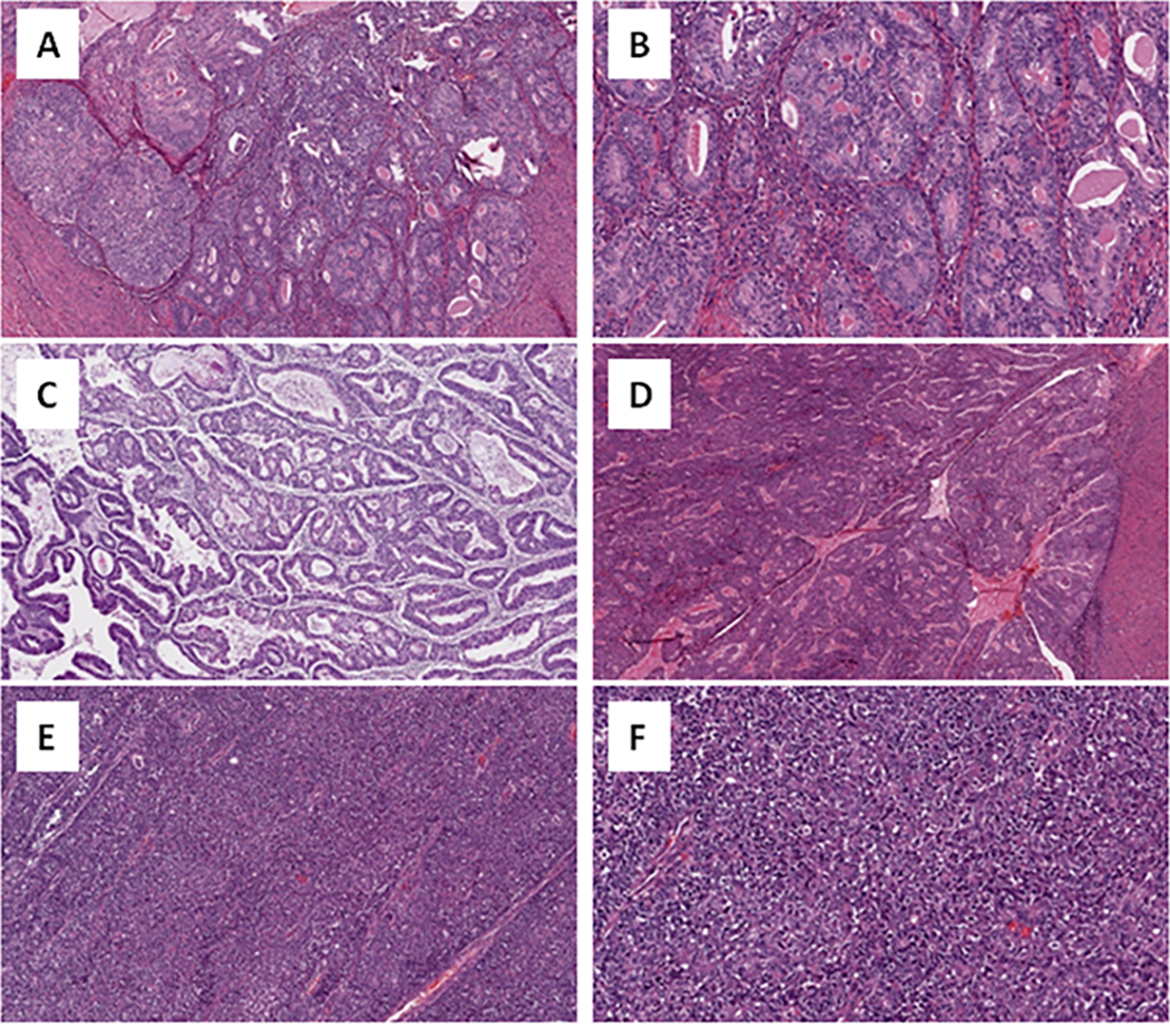
H&E images of representative endometrial endometrioid adenocarcinoma cases containing activating *CCND1* T286I and P287S mutations. A (40x, case D1-A0ZO), B (400x, case D1-A0ZO) and C (40x, case B5-A0JV): grade 1 endometrioid adenocarcinoma containing T286I mutations. D (40x, case D1-A0ZU): grade 1 endometrioid adenocarcinoma containing P287S mutation. E (40x, case D1-A17D) and F (400x, case D1-A17D): grade 3 endometrioid adenocarcinoma containing P287S mutation.

Our analysis of *CCND1* mutations subsequently focused on two endometrial adenocarcinoma cohorts, since this cancer subtype was most frequently enriched for c-terminal cyclin D1 mutations (Fig 1C and 1D). First, we analyzed the primary endometrial adenocarcinoma (EMCA) cohort (n = 248) of the Cancer Genome Atlas Project (TCGA) study [18], in which *CCND1* was mutated in 6.1% (15 of 248) cases (Table 1). In the TCGA EMCA cohort, the majority of *CCND1* mutations (60%, 9 of 15) were located in the carboxy terminal region of cyclin D1 (Fig 1C and Table 3). Next, the pathological data was re-reviewed in terms of patient age, tumor type, stage and FIGO grade. Patient age in *CCND1*-mutated cases ranged from 33 to 76 years (Mean = 56; median = 58) (Table 3). The majority of *CCND1*-mutated cases were of stage I (73%, 11 of 15) and FIGO grade 1 or 2 tumors (40% and 47% respectively) (Table 3). A smaller percentage of *CCND1*-mutated primary TGCA EMCA tumors were stage III (20%, 3 of 15), stage IV (7%, 1 of 15) or FIGO grade 3 (13%, 2 of 15). For all *CCND1*-mutated TGCA EMCA cases, the tumors were of endometrioid histological subtype, which were confirmed by reviewing available H&E images (Fig 2).

**Table 3:** *CCND1* mutations in primary endometrial adenocarcinomas of the TCGA cohort (n = 248) with corresponding patient clinico-pathological characteristics.

n	Sample ID	Mutation	Type	Effect	Age	Histologic Type	Grade	Stage
1	AP-A0LM	E135D	Missense	Unknown	33	Endometrioid	2	III
2	AX-A0J0	A171V	Missense	Unknown	47	Endometrioid	2	I
3	AX-A05Z	F232L	Missense	Unknown	37	Endometrioid	1	III
4	AX-A063	D240N	Missense	Unknown	63	Endometrioid	2	I
5	AP-A054	C243R	Missense	Unknown	64	Endometrioid	2	III
6	BG-A0MQ	L244I	Missense	Unknown	71	Endometrioid	1	I
7	AX-A062	E275*	Nonsense	Likely oncogenic	53	Endometrioid	2	I
8	BS-A0TA	E280del	IF del	Unknown	58	Endometrioid	2	IV
9	B5-A0JV	T286I	Missense	Oncogenic	63	Endometrioid	1	I
10	D1-A0ZO	T286I	Missense	Oncogenic	75	Endometrioid	1	I
11	D1-A0ZU	P287S	Missense	Oncogenic	34	Endometrioid	2	I
12	D1-A17D	P287S	Missense	Oncogenic	58	Endometrioid	3	I
13	BS-A0TJ	R291dup	IF ins	Likely oncogenic	59	Endometrioid	3	I
14	D1-A16D	R291_V293	IF del	Likely oncogenic	49	Endometrioid	1	I
15	BS-A0U5	V293G	Missense	Unknown	76	Endometrioid	1	I

IF del = in frame deletion; FS Ins = frame shift insertion

* = truncating mutations.

The TCGA has previously stratified endometrioid endometrial adenocarcinomas into 4 different molecular subgroups: 1) low copy-number alterations, 2) high copy-number alterations (serous-like), 3) POLE ultramutated and 4) microsatellite instability hypermutated [18]. Within this molecular stratification system, we identified c-terminal cyclin D1 mutations in the low copy-number alterations and in the microsatellite instability hypermutated molecular subgroups (data not shown), both of which have intermediate prognosis. In contrast, no c-terminal cyclin D1 mutations were identified in the POLE ultramutated (best prognosis) and in the copy-number high, serous-like (worst prognosis) molecular subgroups (data not shown) or in TCGA tumors with specific serous-type histology (data not shown).

### *CCND1* mutations in advanced or metastatic endometrial carcinoma

We next interrogated the endometrial carcinoma cohort of cases from the MSK-IMPACT study in order to determine whether *CCND1* c-terminal mutations also occurred in the advanced or metastatic endometrial carcinoma setting [19]. In this cohort of advanced EMCA (n = 210), *CCND1* was mutated in 7% (15 of 210) cases with the majority of mutations (80%, 12 of 15) located in the c-terminus of cyclin D1 (Fig 1D and Table 4). Of the 15 total *CCND1*-mutated cases identified, molecular data was available in 10 metastatic sites, in which the c-terminus of *CCND1* was mutated in 80% (8 of 10) metastatic EMCA sites. While H&E images were not available for re-review, the majority of *CCND1*-mutated MSK-IMPACT advanced or metastatic EMCA cases were reportedly of endometrioid histological subtype (86.7%, 13 of 15). Additionally, one reportedly undifferentiated endometrial carcinoma also contained a c-terminal *CCND1* mutation, while an endometrial serous carcinoma contained an n-terminal cyclin D1 mutation of unknown functional significance (Table 4). The combined overall findings of both TCGA and MSK-IMPACT EMCA cohorts support the notion that *CCND1* c-terminal mutations are enriched in endometrial endometrioid-type carcinomas in both primary and advanced metastatic settings.

**Table 4:** *CCND1* mutations in advanced or metastatic endometrial carcinomas of the MSK-IMPACT cohort (n = 15) with corresponding patient clinico-pathological characteristics and *CCND1* mutation effect.

n	Sample ID	Mutation	Type	Effect	Histologic Type	Site	Status
1	P0008646-T01	T12I	Missense	Unknown	Endometrioid	Abdominal met	Deceased
2	P0000230-T01	N24S	Missense	Unknown	Serous	Primary uterus	Living
3	P0012358-T01	S55F	Missense	Unknown	Endometrioid	Vaginal met	Living
4	P0012397-T01	A171V	Missense	Unknown	Endometrioid	Primary uterus	Living
5	P0012058-T01	E275*	Nonsense	Likely oncogenic	Endometrioid	Primary uterus	Living
6	P0000069-T01	E279*	Nonsense	Likely oncogenic	Endometrioid	Colonic met	Living
7	P0012152-T01	L283_D294del	IF del	Oncogenic	Endometrioid	Peritoneal met	Living
8	P0001248-T01	T286I	Missense	Oncogenic	Endometrioid	Lymph node met	Living
9	P0006849-T01	T286I	Missense	Oncogenic	Undifferentiated	Primary uterus	Living
10	P0009518-T01	T286I	Missense	Oncogenic	Endometrioid	Muscle met	Living
11	P0010310-T01	T286I	Missense	Oncogenic	Endometrioid	Primary uterus	Deceased
12	P0003767-T01	P287A	Missense	Oncogenic	Endometrioid	Omental met	Living
13	P0011569-T01	P287S	Missense	Oncogenic	Endometrioid	Primary uterus	Living
14	P0005285-T01	D289del	IF del	Oncogenic	Endometrioid	Pelvic met	Deceased
15	P0005285-T02	D289del	IF del	Oncogenic	Endometrioid	Primary uterus	Deceased
16	P0008444-T01	V290Afs*66	FS Ins	Likely oncogenic	Endometrioid	Lung met	Deceased
17	P0008646-T01	V293del	IF del	Likely oncogenic	Endometrioid	Abdominal met	Deceased

Note: one tumor (P0008646-T01) had more than 1 *CCND1* mutation, and one patient (P0005285) had two samples analyzed (primary and metastatic tumors). IF del = in frame deletion; FS Ins = frame shift insertion

* = truncating mutations.

### Functional effect of c-terminal *CCND1* mutations in endometrial cancer

The majority of *CCND1*-mutated endometrial cancer cases (70%, 21 of 30 endometrial cancers, Tables 3 and 4) contained mutations in the c-terminus of cyclin D1 that may be implicated in cyclin D1 activation and gain-of-function (Tables 3 and 4). Recurrent activating mutations in endometrial cancer included: E275*, T286I (most common), P287S (second most common), D289del (Fig 1C and 1D and Tables 3 and 4). Less frequent mutations in endometrial cancer that are also likely oncogenic included: E279*, L283_294del, V290Afs*, R291dup, R291_V293del and V293del (Tables 3 and 4). Based on prior *in vitro* and *in vivo* studies, these mutations are predicted to drive cellular transformation and activation of CDK4/6 by disrupting cyclin D1 Thr-286 phosphorylation, nuclear export and proteasome-mediated degradation [12][10][11]. These findings suggest that a subset endometrial adenocarcinomas possesses a unique spectrum of somatic *CCND1* mutations in which activating mutations occurring in the carboxy terminal domain of cyclin D1 are enriched, compared to other cancer types, thereby promoting cyclin D1 nuclear expression, gain of function and oncogenic activation.

### Co-occurring mutations in endometrial cancer

TCGA cases exhibiting C-terminal *CCND1* mutations had frequent co-occurring mutations of genes implicated in the pathogenesis of endometrial endometrioid adenocarcinomas, as the majority of *CCND1*-mutated cases exhibited mutations of *ARID1A* and members of the PI3K signaling pathway, *PTEN* and *PIK3CA*. Less frequent co-occurring mutations were seen in other genes involved in endometrial carcinogenesis such as *KRAS*, *CTNNB1*, *ARID5B* and *PMS2* in a minority of *CCND1*-mutated cases. In contrast, there were no co-occurring mutations of *TP53*, which is frequently mutated in endometrial serous and serous-like carcinomas, or of *POLE*, which is altered in ultramutated endometrial carcinomas, or of *MSH2*, *MSH6* and *MLH1*, which are mutated in microsatellite unstable endometrial carcinomas (S2 Fig).

### C-terminal *CCND1* mutations in other cancer types

Although occurring less frequently than in endometrial cancer, c-terminal activating *CCND1* mutations were also identified in other cancer types, such as gastrointestinal adenocarcinoma (esophageal, stomach, colorectal, ampula of vater), squamous cell carcinoma (head & neck, lung, skin), urothelial carcinoma, cutaneous melanoma, angiosarcoma, ovarian carcinoma (low grade serous and endometrioid types) and uterine epithelioid leiomyosarcoma (Table 5). The most common activating mutations occurred in amino acids P287 and T286 as missense mutations, or as nonsense mutations in amino acids Q261, E275, E279, E280, E285, all of which are predicted to directly disrupt the phospho-degron site and/or nuclear export signal of cyclin D1, thereby resulting in nuclear accumulation and protein overexpression. The rate of overall and c-terminal activating *CCND1* mutations in these non-endometrial carcinoma types was rare, at most approximately 1% in melanoma and colorectal carcinoma. The overall rate of *CCND1* mutations was 1.1% in colorectal carcinoma and cutaneous melanoma (23 of 2,067 cases and 8 of 708 cases respectively) with a smaller percentage of mutations occurring specifically in the c-terminal region of cyclin D1 (0.7%, 5 of 708 cases in cutaneous melanoma and 0.4%, 9 of 2067 in colorectal carcinoma).

**Table 5:** C-terminal *CCND1* mutations across other non-endometrial carcinoma and non-mantle cell lymphoma cancer types.

Study	Sample ID	Cancer Type	Mutation	Effect	Type
Ampullary Carcinoma (Baylor, Cell Rep 2016)	DUOAC_781	Ampulla of Vater	Q261*	Likely oncogenic	Nonsense
MSK-IMPACT (MSKCC, Nat Med 2017)	P-0000148-T01-IM3	LG serous ovarian CA	Q261*	Likely oncogenic	Nonsense
MSK-IMPACT (MSKCC, Nat Med 2017)	P-0003765-T01-IM5	Angiosarcoma	Q264*	Likely oncogenic	Nonsense
Melanoma (Broad/Dana Farber, Nature 2012)	ME049	Melanoma	A270T	Unknown	Missense
Pan-Lung Cancer (TCGA, Nat Genet 2016)	TCGA-44-3396-01	Lung Adenocarcinoma	A271S	Unknown	Missense
Esophagus-Stomach Cancers (TCGA, Nat 2017)	TCGA-BR-4363-01	Stomach ACA	A271T	Unknown	Missense
Pan-Lung Cancer (TCGA, Nat Genet 2016)	TCGA-51-6867-01	Lung SCC	E275*	Likely oncogenic	Nonsense
MSK-IMPACT (MSKCC, Nat Med 2017)	P-0012241-T02-IM5	Lung SCC	E276V	Unknown	Missense
Colorectal ACA (DFCI, Cell Reports 2016)	dfci_2016_3091	Colorectal ACA	E278G	Unknown	Missense
MSK-IMPACT (MSKCC, Nat Med 2017)	P-0004495-T01-IM5	Lung SCC	E279*	Likely oncogenic	Nonsense
Bladder Urothelial Carcinoma (TCGA, Nat 2014)	TCGA-DK-A1A6-01	Bladder urothelial CA	E280del	Unknown	IF_Del
Esophagus-Stomach Cancers (TCGA, Nat 2017)	TCGA-KH-A6WC-01	Esophageal SCC	E280del	Unknown	IF_Del
Melanoma tumors (UCLA, Cell 2016)	Pt11	Cutaneous melanoma	E280*	Likely oncogenic	Nonsense
Colorectal ACA (DFCI, Cell Reports 2016)	dfci_2016_3064	Colorectal ACA	E280V	Unknown	Missense
H&N SCC (TCGA, Nature 2015)	TCGA-CV-6441-01	Head &Neck SCC	D282H	Unknown	Missense
Colorectal ACA (DFCI, Cell Reports 2016)	dfci_2016_111	Colorectal ACA	A284V	Unknown	Missense
Esophagus-Stomach Cancers (TCGA, Nat 2017)	TCGA-L5-A8NM-01	Esophageal ACA	C285*	Likely oncogenic	Nonsense
H&N SCC (TCGA, Nature 2015)	TCGA-CV-6441-01	Head & Neck SCC	C285_P287del	Likely oncogenic	IF_Del
MSK-IMPACT (MSKCC, Nat Med 2017)	P-0011446-T01-IM5	Colorectal ACA	T286A	Oncogenic	Missense
Colorectal ACA (DFCI, Cell Reports 2016)	dfci_2016_2624	Colorectal ACA	T286I	Oncogenic	Missense
Colorectal ACA (DFCI, Cell Reports 2016)	dfci_2016_3319	Colorectal ACA	T286I	Oncogenic	Missense
MSK-IMPACT (MSKCC, Nat Med 2017)	P-0005364-T01-IM5	Up. tract urothelial CA	T286I	Oncogenic	Missense
Pan-Lung Cancer (TCGA, Nat Genet 2016)	TCGA-85-8072-01	Lung SCC	P287A	Oncogenic	Missense
Melanoma (Broad/Dana Farber, Nature 2012)	ME024	Melanoma	P287L	Oncogenic	Missense
MSK-IMPACT (MSKCC, Nat Med 2017)	P-0000396-T01-IM3	Oropharynx SCC	P287L	Oncogenic	Missense
Skin SCC (DFCI, Clin Cancer Res 2015)	TP-S07-16280-NT	Cutaneous SCC	P287L	Oncogenic	Missense
Cancer Cell Line Encyclopedia (Broad, Nat 2012)	OVK18_OVARY	Ovary endometrioid CA	P287R	Oncogenic	Missense
MSK-IMPACT (MSKCC, Nat Med 2017)	P-0001821-T01-IM3	Uterine epithelioid LMS	P287S	Oncogenic	Missense
MSK-IMPACT (MSKCC, Nat Med 2017)	P-0005455-T01-IM5	Colon ACA	P287S	Oncogenic	Missense
Colorectal ACA (DFCI, Cell Reports 2016)	dfci_2016_2564	Colorectal ACA	R291W	Unknown	Missense
MSK-IMPACT (MSKCC, Nat Med 2017)	P-0001685-T01-IM3	Rectal ACA	D292G	Unknown	Missense

Note: IF_del = in frame deletion

* = truncating mutations.

CA = carcinoma; ACA = adenocarcinoma; SCC = squamous cell carcinoma; LMS = leiomyosarcoma.

## Discussion

Cyclin D1, encoded by the *CCND1* gene, is a core cell cycle protein that promotes cellular proliferation by activating CDK4/6 kinases and progression of the G1-S phase of cell cycle. *CCND1* acts as oncogene and is frequently overexpressed in a variety of cancers often via gene amplification or gene rearrangement [23][24][25]. Recently, *CCND1* mutations inhibiting Thr286 phosphorylation, nuclear export and ubiquitin-mediated degradation by the proteasome have been shown to result in constitutive activation of CDK4/6 complexes, thereby promoting cellular proliferation and malignant transformation both *in vitro* and *in vivo* [6][7][8]. Corroborating its oncogenic potential, *CCND1* mutations affecting Thr286 phosphorylation and inhibiting nuclear export have been reported in a few cancers [10][12][11]; however, the frequency and spectrum of *CCND1* mutations have not been systematically investigated or reported across a large series of cases and across a variety of cancer subtypes. We exploited and mined the publicly available data of the Cancer Genome Atlas Project and other published exome sequencing studies to interrogate the frequency, spectrum and oncogenic effects of *CCND1* mutations across of a large number and diverse set of human cancers.

Here, we demonstrate that *CCND1* mutations are of very low frequency across the vast majority of cancer types (0.5%, 151 of 29,432 cases), but are predominantly enriched in endometrial endometrioid carcinomas in both the primary and metastatic settings (6.5%, 30 of 458 cases). In endometrial cancer, the majority of *CCND*1 mutations were located in the carboxy-terminal region of cyclin D1 (70%, 21 of 30 endometrial cancers), likely resulting in cyclin D1 oncogenic activation of cyclin D1-CDK4/6 complexes and gain of function. To our knowledge, this is the first study to systematically evaluate and report activating c-terminal *CCND1* mutations across a large number of diverse cancer types. Our results are in line with two other previously published studies in which oncogenic, c-terminal cyclin D1 mutations were identified in endometrial cancer [10][11]. Ikeda et al identified *CCND1* mutations in 2.3% (2 of 88) of endometrial cancer cases. Their two reported mutated cases contained a T286I mutation, which resulted in constitutive cyclin D1 nuclear accumulation and oncogenic activation [11]. Likewise, in an independent endometrial cancer cohort, Moreno-Bueno et al identified *CCND1* mutations in 2.5% (3 of 119) endometrial cancer cases. Two cases demonstrated mutation at amino acid P287 (P287S and P287T), while a third case contained a 12-bp in-frame deletion that eliminated amino acids 289–292, all of which resulted in cyclin D1 nuclear expression in more than 50% of endometrial cancer cells [10]. Importantly, Benzeno et al independently confirmed that *CCND1* mutations at T286, P287, D289 or deletion of amino acids 289–292 resulted in cyclin D1 nuclear retention, activation of CDK4/6 kinase and cellular transformation [12]. The rate of overall *CCND1* mutations in endometrial cancer in our study is higher than that reported by Ikeda et al and Moreno-Bueno et al, likely reflecting the fact that the two prior studies sequenced a target region of cyclin D1 as opposed to next generation sequencing of the whole *CCND1* gene. Our study expands the findings of Moreno-Bueno et al and Ikeda et al [10][11], in which *CCND1* mutations at amino acids T286, P287, D289, deletion of amino acids 289–292 and potentially other c-terminal CCND1 mutations, such as nonsense mutation at E275*, E279*, may be important in the pathogenesis of a subset of endometrial cancers.

Importantly, the majority of TCGA cases exhibiting c-terminal *CCND1* mutations had frequent co-occurring mutations of *ARID1A* and members of the PI3K signaling pathway, *PTEN* and *PIK3CA*, suggesting that alterations these molecular pathways are also required in addition to oncogenic nuclear cyclin D1 activation in a subset of endometrial cancers. In addition, a search of the BROAD Institute gene variant database–derived from whole exomes of healthy individuals from many ethnic or geographic settings did not reveal any of the oncogenic c-terminal *CCND1* mutations or polymorphisms indentified in our study in a control population that did not harbor cancer.

Our results also corroborate the findings of Chang et al, who identified Proline 287 as a recurrent hotspot mutated amino acid of cyclin D1 in a population-scale cohort of tumor samples of various cancer types [26]. Consistent with the findings of Chang et al, Proline 287 was the most commonly mutated amino acid of cyclin D1 across all cancer types in our study. We identified mutations in cyclin D1 at Proline 287 in a diverse tumor set including melanoma, endometrial carcinoma, colonic adenocarcinoma, ovarian endometrioid adenocarcinoma, uterine leyomyosarcoma and squamous cell carcinoma of lung, oro-pharynx and skin, thus supporting the notion that *CCND1* driver mutations at Pro287 may be a recurrent hotspot in cancer.

In addition, we also demonstrate high frequency of oncogenic *CCND1* mutations at or surrounding amino acid Threonine 286 in the c-terminus and at or surrounding Tyrosine 44 at the n-terminus, albeit in different tumor types (i.e. carcinoma, particularly endometrial, and mantle cell lymphoma respectively). Of note, in mantle cell lymphoma, it has also been previously demonstrated that, compared to wild type *CCND1*, n-terminal E36K, Y44D or C47S *CCND1* mutations are likely oncogenic and increased cyclin D1 protein levels through attenuation of threonine-286 phosphorylation, thereby promoting resistance to ibrutinib, which is an FDA-approved Bruton tyrosine kinase inhibitor for mantle cell lymphoma treatment [27]. Future studies are needed in order to determine whether patients, whose tumors contain oncogenic activating mutations of *CCND1*, would benefit from precision medicine by targeting cyclin D1-CDK4/6 kinase complexes with specific inhibitors.

In the past decade, the genomic landscape of many different types of tumors have been studied and deposited in publicly available data portals [28]. In addition, the clinical sequencing of advanced, metastatic tumors is becoming a mainstay in cancer care to guide potential treatment in mutation- and target-specific precision medicine [19]. In both clinical and preclinical research settings, a host of tools and resources can be used to interrogate specific genetic events relative to clinico-pathological characteristics of patient tumors as well as to generate hypotheses for future testing. Currently, the treatment options for advanced endometrial cancer patients are limited, and there is scant data with regards to the efficacy of palbociclib, a CDK4/6 specific inhibitor, towards endometrial cancer. In preclinical models, palbociclib or knockout of cyclin D1 had therapeutic potential against endometrial cancer cell lines and xenografts expressing the retinoblastoma protein, Rb [29][30]. Lastly, our findings implicate oncogenic c-terminal mutations of *CCND1* in the pathogenesis of a subset of human cancers and provide a key resource to guide future preclinical and clinical investigations, especially with regards to the use of palbociclib or similar CDK4/6 specific inhibitors in a subset of endometrial cancers.

## Supporting information

S1 Table List of exome studies with respective cancer types and sites of origin that were mined for *CCND1* mutations. (PDF)

S2 Table *CCND1* mutations in the mantle cell lymphoma IBIDIPS cohort. IF del = in frame deletion; FS Ins = frame shift insertion; * = truncating mutations.
(PDF)

S1 Fig Diagrams of *CCND1* mutations in multiple myeloma (A), in colorectal adenocarcinoma (B) and in cutaneous melanoma (C).
(PDF)

S2 Fig Diagram of co-occurring mutations of genes frequently implicated in endometrial cancer in the TCGA cohort of endometrial carcinoma cases exhibiting *CCND1* mutations. Square denotes presence of gene mutation. Red bar denotes presence of c-terminal *CCND1* mutation.
(PDF)
