# Supplementary material for: Oncogenic c-terminal cyclin D1 (CCND1) mutations are enriched in endometrioid endometrial adenocarcinomas
Source: PLoS One. 2018 Jul 3;13(7):e0199688. doi: 10.1371/journal.pone.0199688 (PMC6029777; doi:10.1371/journal.pone.0199688)
Supplement: S1 Table — (PDF) [file pone.0199688.s001.pdf]

List of Studies Analyzed for Cyclin D1 Mutations
Acinar Cell Carcinoma of the Pancreas (Johns Hopkins, J Pathol 2014)
Acute Myeloid Leukemia (TCGA, NEJM 2013)
Adenoid Cystic Carcinoma (FMI, Am J Surg Pathl. 2014)
Adenoid Cystic Carcinoma (MDA, Clin Cancer Res 2015)
Adenoid Cystic Carcinoma (MSKCC, Nat Genet 2013)
Adenoid Cystic Carcinoma (Sanger/MDA, JCI 2013)
Adenoid Cystic Carcinoma of the Breast (MSKCC, J Pathol. 2015)
Ampullary Carcinoma (Baylor College of Medicine, Cell Reports 2016)
Bladder Cancer (MSKCC, Eur Urol 2014)
Bladder Cancer (MSKCC, JCO 2013)
Bladder Cancer, Plasmacytoid Variant (MSKCC, Nat Genet 2016)
Bladder Urothelial Carcinoma (BGI, Nat Genet 2013)
Bladder Urothelial Carcinoma (Dana Farber & MSKCC, Cancer Discov 2014)
Bladder Urothelial Carcinoma (TCGA, Nature 2014)
Breast Cancer (METABRIC, Nature 2012 & Nat Commun 2016)
Breast cancer patient xenografts (British Columbia, Nature 2014)
Breast Invasive Carcinoma (British Columbia, Nature 2012)
Breast Invasive Carcinoma (Broad, Nature 2012)
Breast Invasive Carcinoma (Sanger, Nature 2012)
Breast Invasive Carcinoma (TCGA, Cell 2015)
Cancer Cell Line Encyclopedia (Novartis/Broad, Nature 2012)
Cholangiocarcinoma (National Cancer Centre of Singapore, Nat Genet 2013)
Cholangiocarcinoma (National University of Singapore, Nat Genet 2012)
Chronic Lymphocytic Leukemia (Broad, Cell 2013)
Chronic Lymphocytic Leukemia (IUOPA, Nature 2015)
Clear Cell Renal Cell Carcinoma (U Tokyo, Nat Genet 2013)
Colorectal Adenocarcinoma (DFCI, Cell Reports 2016)
Colorectal Adenocarcinoma (Genentech, Nature 2012)
Colorectal Adenocarcinoma (TCGA, Nature 2012)
Colorectal Adenocarcinoma Triplets (MSKCC, Genome Biol 2014)
Cutaneous squamous cell carcinoma (DFCI, Clin Cancer Res 2015)
Cutaneous T Cell Lymphoma (Columbia U, Nat Genet 2015)
Cystic Tumor of the Pancreas (Johns Hopkins, PNAS 2011
Desmoplastic Melanoma (Broad Institute, Nat Genet 2015)
Diffuse Large B-Cell Lymphoma (Broad, PNAS 2012)
Esophageal Adenocarcinoma (Broad, Nat Genet 2013)
Esophageal Squamous Cell Carcinoma (ICGC, Nature 2014)
Esophageal Squamous Cell Carcinoma (UCLA, Nat Genet 2014)
Ewing Sarcoma (Institut Cuire, Cancer Discov 2014)
Gallbladder Carcinoma (Shanghai, Nat Genet 2014)
Gastric Adenocarcinoma (TMUCIH, PNAS 2015)
Genetic Characterization of NSCLC young adult patients (University of Turin, Lung Cancer 2016)
Genomic Hallmarks of Prostate Adenocarcinoma (CPC-GENE, Nature 2017)

Glioblastoma (TCGA, Cell 2013)
Head and Neck Squamous Cell Carcinoma (Broad, Science 2011)
Head and Neck Squamous Cell Carcinoma (Johns Hopkins, Science 2011)
Head and Neck Squamous Cell Carcinoma (TCGA, Nature 2015)
Hepatocellular Adenoma (Inserm, Cancer Cell 2014)
Hepatocellular Carcinomas (Inserm, Nat Genet 2015)
Hypodiploid Acute Lymphoid Leukemia (St Jude, Nat Genet 2013)
Infant MLL-Rearranged Acute Lymphoblastic Leukemia (St Jude, Nat Genet 2015)
Insulinoma (Shanghai, Nat Commun 2013)
Intrahepatic Cholangiocarcinoma (Johns Hopkins University, Nat Genet 2013)
Kidney Chromophobe (TCGA, Cancer Cell 2014)
Kidney Renal Clear Cell Carcinoma (BGI, Nat Genet 2012)
Kidney Renal Clear Cell Carcinoma (TCGA, Nature 2013)
Liver Hepatocellular Carcinoma (AMC, Hepatology 2014)
Liver Hepatocellular Carcinoma (RIKEN, Nat Genet 2012)
Low-Grade Gliomas (UCSF, Science 2014).
Lung Adenocarcinoma (Broad, Cell 2012)
Lung Adenocarcinoma (MSKCC 2015)
Lung Adenocarcinoma (TCGA, Nature 2014)
Lung Adenocarcinoma (TSP, Nature 2008)
Lung Squamous Cell Carcinoma (TCGA, Nature 2012)
Malignant Peripheral Nerve Sheath Tumor (MSKCC, Nat Genet 2014)
Malignant Pleural Mesothelioma (NYU, Cancer Res 2015)
Mantle Cell Lymphoma (IDIBIPS, PNAS 2013)
Medulloblastoma (Broad, Nature 2012)
Medulloblastoma (ICGC, Nature 2012)
Medulloblastoma (PCGP, Nature 2012)
Medulloblastoma (Sickkids, Nature 2016)
Melanoma (Broad/Dana Farber, Nature 2012)
Merged Cohort of LGG and GBM (TCGA, Cell 2016)
Metastatic Prostate Cancer, SU2C/PCF Dream Team (Robinson et al., Cell 2015)
MSK-IMPACT Clinical Sequencing Cohort (MSKCC, Nat Med 2017)
Multiple Myeloma (Broad, Cancer Cell 2014)
Multiregion Sequencing of Clear Cell Renal Cell Carcinoma (IRC, Nat Genet 2014)
Mutational profiles of metastatic breast cancer (France, 2016)
Myelodysplasia (Tokyo, Nature 2011)
Nasopharyngeal Carcinoma (Singapore, Nat Genet 2014)
NCI-60 Cell Lines (NCI, Cancer Res. 2012)
Neuroblastoma (AMC Amsterdam, Nature 2012)
Neuroblastoma (Broad, Nat Genet 2013)
Neuroendocrine Prostate Cancer (Trento/Cornell/Broad 2016)
NGS in Anaplastic Oligodendroglioma and Anaplastic Oligoastrocytomas tumors (MSK, Neuro Oncol 2017
Oral Squamous Cell Carcinoma (MD Anderson, Cancer Discov 2013)
Ovarian Serous Cystadenocarcinoma (TCGA, Nature 2011)

Paired-exome sequencing of acral melanoma (TGEN, Genome Res 2017)
Pancreatic Adenocarcinoma (ICGC, Nature 2012)
Pancreatic Adenocarcinoma (QCMG, Nature 2016)
Pancreatic Cancer (UTSW, Nat Commun 2015)
Pancreatic Neuroendocrine Tumors (Johns Hopkins University, Science 2011)
Pancreatic Neuroendocrine Tumors (Nature, 2017)
Pan-Lung Cancer (TCGA, Nat Genet 2016)
Papillary Thyroid Carcinoma (TCGA, Cell 2014)
Pediatric Ewing Sarcoma (DFCI, Cancer Discov 2014)
Pilocytic astrocytomasatic (Nat Genetics, 2013)
Primary Central Nervous System Lymphoma (Mayo Clinic, Clin Cancer Res 2015)
Prostate Adenocarcinoma (Broad/Cornell, Cell 2013)
Prostate Adenocarcinoma (Broad/Cornell, Nat Genet 2012)
Prostate Adenocarcinoma (Fred Hutchinson CRC, Nat Med 2016)
Prostate Adenocarcinoma (MSKCC, Cancer Cell 2010)
Prostate Adenocarcinoma (TCGA, Cell 2015)
Prostate Adenocarcinoma CNA study (MSKCC, PNAS 2014)
Prostate Adenocarcinoma, Metastatic (Michigan, Nature 2012)
Renal Non-Clear Cell Carcinoma (Genentech, Nat Genet 2014)
Rhabdomyosarcoma (NIH, Cancer Discov 2014)
Sarcoma (MSKCC/Broad, Nat Genet 2010)
Skin Cutaneous Melanoma (Broad, Cell 2012)
Skin Cutaneous Melanoma (Yale, Nat Genet 2012)
Skin cutaneous melanoma tumors (UCLA, Cell 2016)
Small Cell Carcinoma of the Ovary (MSKCC, Nat Genet 2014)
Small Cell Lung Cancer (CLCGP, Nat Genet 2012)
Small Cell Lung Cancer (Johns Hopkins, Nat Genet 2012)
Small Cell Lung Cancer (U Cologne, Nature 2015)
Stomach Adenocarcinoma (Pfizer and UHK, Nat Genet 2014)
Stomach Adenocarcinoma (TCGA, Nature 2014)
Stomach Adenocarcinoma (U Tokyo, Nat Genet 2014)
Stomach Adenocarcinoma (UHK, Nat Genet 2011)
Targeted gene sequencing in 62 high-grade primary Unclassified Renal Cell Carcinoma (MSK, Nature 201[illegible]
TCGA data for Esophagus-Stomach Cancers (TCGA, Nature 2017)
Thymic Epithelial Tumors (NCI, Nat Genet 2014)
Uterine Carcinosarcoma (Johns Hopkins University, Nat Commun 2014)
Uterine Corpus Endometrial Carcinoma (TCGA, Nature 2013)
