# Supplementary material for: Oncogenic c-terminal cyclin D1 (CCND1) mutations are enriched in endometrioid endometrial adenocarcinomas
Source: PLoS One. 2018 Jul 3;13(7):e0199688. doi: 10.1371/journal.pone.0199688 (PMC6029777; doi:10.1371/journal.pone.0199688)
Supplement: S2 Table — IF del = in frame deletion; FS Ins = frame shift insertion; * = truncating mutations. (PDF) [file pone.0199688.s002.pdf]

Supplementary Table 2

Sample ID	Cancer Type	Protein Change	Functional Impact	Mutation Type
M027	Mantle Cell Lymphoma	S41T	Unknown	Missense
M027	Mantle Cell Lymphoma	V42E	Unknown	Missense
M004	Mantle Cell Lymphoma	Y44*	Likely oncogenic	Nonsense
M003	Mantle Cell Lymphoma	Y44C	Likely oncogenic	Missense
M015	Mantle Cell Lymphoma	Y44D	Likely oncogenic	Missense
M009	Mantle Cell Lymphoma	Y44D	Likely oncogenic	Missense
M004	Mantle Cell Lymphoma	Y44H	Likely oncogenic	Missense
M011	Mantle Cell Lymphoma	Y44S	Likely oncogenic	Missense
M021	Mantle Cell Lymphoma	K46E	Unknown	Missense
M018	Mantle Cell Lymphoma	C47S	Likely oncogenic	Missense
M002	Mantle Cell Lymphoma	C47S	Likely oncogenic	Missense
M022	Mantle Cell Lymphoma	V290G	Unknown	Missense

Supplemental table 2. *CCND1* mutations in the mantle cell lymphoma IDIBIPS study cohort. IF del= in frame deletion; FS Ins=frame shift insertion; *= truncating mutations. Note: 2 tumors had more than mutation.
