# Supplementary material for: Oncogenic c-terminal cyclin D1 (CCND1) mutations are enriched in endometrioid endometrial adenocarcinomas
Source: PLoS One. 2018 Jul 3;13(7):e0199688. doi: 10.1371/journal.pone.0199688 (PMC6029777; doi:10.1371/journal.pone.0199688)
Supplement: S1 Fig — Diagrams of CCND1 mutations in multiple myeloma (A), in colorectal adenocarcinoma (B) and in cutaneous melanoma (C). (PDF) [file pone.0199688.s003.pdf]

Supplementary Figure 1

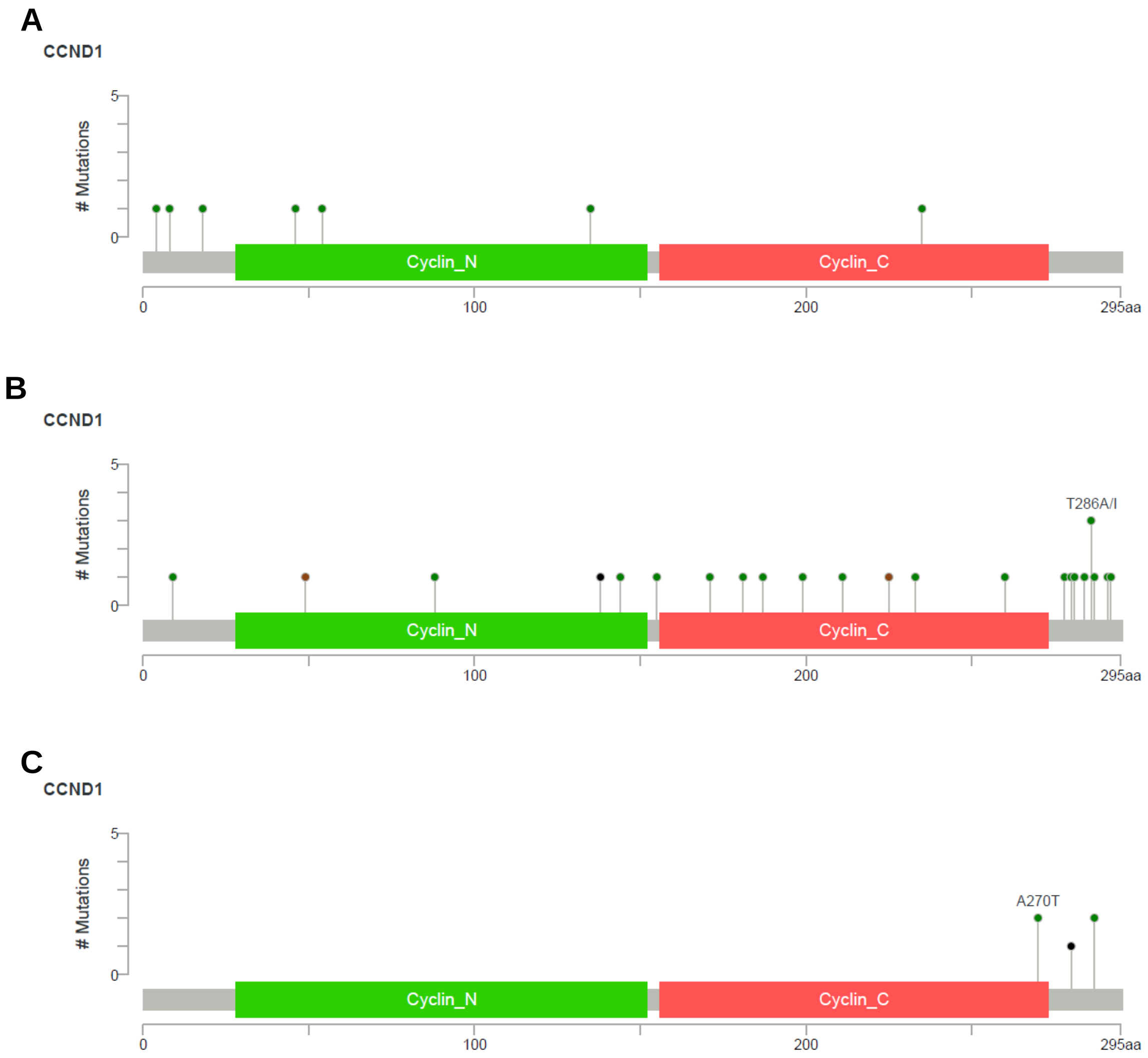

Supplemental Figure 1. Diagrams of *CCND1* mutations in multiple myeloma (A), in colorectal adenocarcinoma (B) and in cutaneous melanoma (C).
