# Supplementary material for: Oncogenic c-terminal cyclin D1 (CCND1) mutations are enriched in endometrioid endometrial adenocarcinomas
Source: PLoS One. 2018 Jul 3;13(7):e0199688. doi: 10.1371/journal.pone.0199688 (PMC6029777; doi:10.1371/journal.pone.0199688)
Supplement: S2 Fig — Square denotes presence of gene mutation. Red bar denotes presence of c-terminal CCND1 mutation. (PDF) [file pone.0199688.s004.pdf]

Supplementary Figure 2

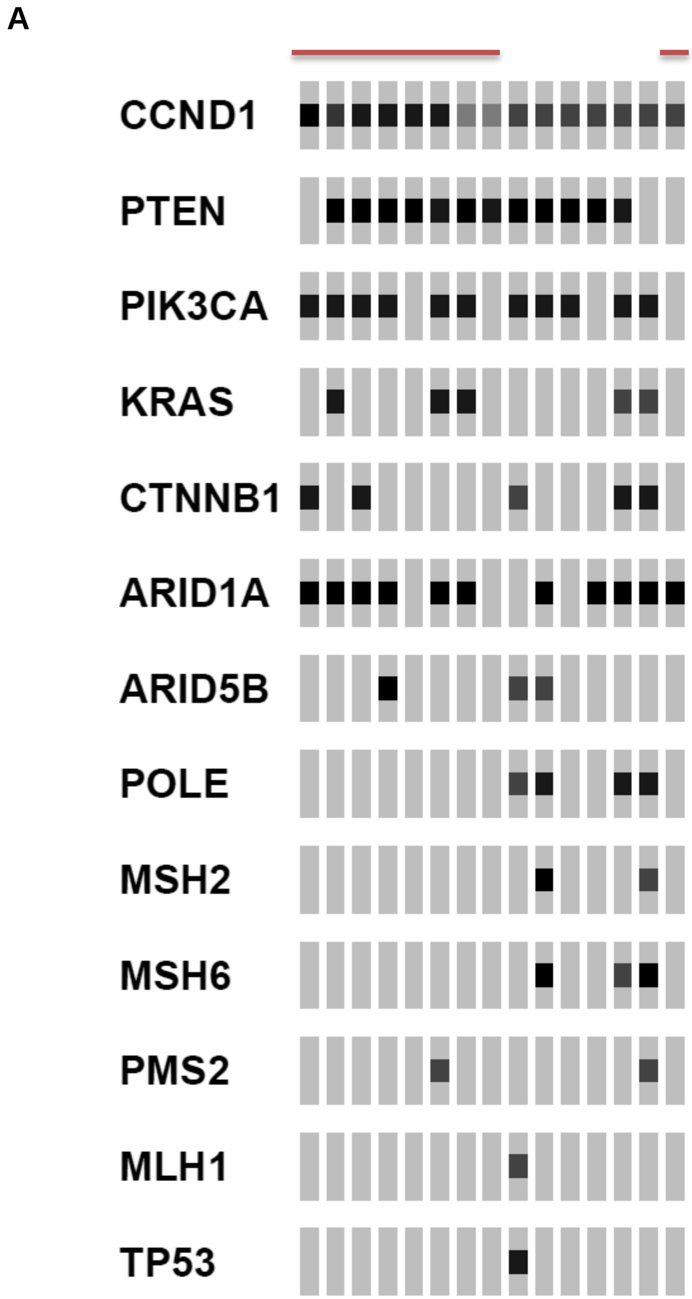

Supplemental Figure 2. Diagram of co-occurring mutations of genes frequently implicated in endometrial cancer in the TCGA cohort of endometrial carcinoma cases exhibiting *CCND1* mutations. Square denotes presence of gene mutation. Red bar denotes presence of C-terminal *CCND1* mutation.
